# 20 years of liver transplantation in Santiago de compostela (Spain). experience review

**DOI:** 10.1186/2197-425X-3-S1-A698

**Published:** 2015-10-01

**Authors:** JR Fernández Villanueva, AM López Lago, R Fernández Garda, S Tomé Martínez de Rituerto, E Varo Pérez

**Affiliations:** Complexo Hospitalario de Santiago de Compostela, Servicio de Medicina Intensiva, Santiago de Compostela, Spain; Complexo Hospitalario de Santiago de Compostela, Unidad de Trasplante Abdominal, Santiago de Compostela, Spain

809 Liver Trasplantations (LT) were made at Hospital Clínico Universitario de Santiago de Compostela (Spain) in the period 1994-2014, accounting 4.25% of all LT performed in Spain (19005 cases, according to the Spanish Liver Transplant Registry- RETH). 20 years of experience review and comparison of Acumulated Surveillance Rates of our experience respect to the Spanish data provided by RETH.

## Materials and Methods

Retrospective and descriptive study of 809 cases of LT performed from 1994 to 2014 at Hospital Clínico Universitario de Santiago de Compostela (Spain) based in our local LT Registration and in the National Spanish Registration- RETH.

## Results

809 LT cases, 12 of which were Hepatorenal Transplantations, 3 required a Re-Transplantation. Media of LT: 36 LT/year. Gender: 79.35% were Male and 20.64% Female with a Mean Age of 51 years old. Blood Group Predominancy: A positive (49%) followed by the group 0 positive (39%). The most frequent indication of TL was: Alcohol-related Cirrhosis (43%), followed by Idiopathic (43.01%) and Fulminant Hepatic Failure (6.18%). The most common indication for Re-Transplantation was Hepatic Artery Thrombosis (34%) followed by Primary Allograft Dysfunction (28.57%). Mortality at 20 years: 34.86%, the most frequent cause of Death was: Recurrence of Underlying Disease (28% with a 80% rate in Hepatitis C Virus Cirrhosis) followed by Bacterial/Fungal infections (20%). The Acumulated Surveillance Rate at 20 years is 47% in our series, higher in comparison with the overall data provided by RETH (37% using Kaplan-Meier Curve *p < 0.01*) and in the last five years (2008-2013) is 77%, higher compared with RETH data for the same period of time (70% *p < 0.05*). The Acumulated Surveillance Rate at 20 years based on LT Indication: 70% in Fulminant Hepatic Failure group, 50% in the Alcoholic Cirrhosis group and 35% in Hepatitis C Virus Cirrhosis. In Re-transplantation group, Acumulated Surveillance Rate at five years (2008-2013) is just 23%.

## Conclusions

Alcohol-related Liver Disease, Hepatitis C Virus and Hepatocellular Carcinoma are the most frequent Indications of LT in the current Transplantation Program. Acumulated Sirveillance Rates are similar to the ones found in other Transplantation Programs. The main Cause of Death is Recurrence of the Underlying Disease, followed by Infectious and Cardiovascular Diseases. High Mortality Rates in Re-transplantation Group leads to a more careful selection of cases.

